# A statistical analysis of cervical auscultation signals from adults with unsafe airway protection

**DOI:** 10.1186/s12984-015-0110-9

**Published:** 2016-01-22

**Authors:** Joshua M. Dudik, Atsuko Kurosu, James L. Coyle, Ervin Sejdić

**Affiliations:** Department of Electrical and Computer Engineering, Swanson School of Engineering, University of Pittsburgh, 3700 O’Hara Street, Pittsburgh, PA, 15261 USA; Department of Communication Science and Disorders, School of Health and Rehabilitation Sciences, University of Pittsburgh, 4028 Forbes Tower, Pittsburgh, PA, 15260 USA

**Keywords:** Cervical auscultation, Aspiration, Dysphagia, Swallow accelerometry, Swallow sounds

## Abstract

**Background:**

Aspiration, where food or liquid is allowed to enter the larynx during a swallow, is recognized as the most clinically salient feature of oropharyngeal dysphagia. This event can lead to short-term harm via airway obstruction or more long-term effects such as pneumonia. In order to non-invasively identify this event using high resolution cervical auscultation there is a need to characterize cervical auscultation signals from subjects with dysphagia who aspirate.

**Methods:**

In this study, we collected swallowing sound and vibration data from 76 adults (50 men, 26 women, mean age 62) who underwent a routine videofluoroscopy swallowing examination. The analysis was limited to swallows of liquid with either thin (<5 cps) or viscous (≈300 cps) consistency and was divided into those with deep laryngeal penetration or aspiration (unsafe airway protection), and those with either shallow or no laryngeal penetration (safe airway protection), using a standardized scale. After calculating a selection of time, frequency, and time-frequency features for each swallow, the safe and unsafe categories were compared using Wilcoxon rank-sum statistical tests.

**Results:**

Our analysis found that few of our chosen features varied in magnitude between safe and unsafe swallows with thin swallows demonstrating no statistical variation. We also supported our past findings with regard to the effects of sex and the presence or absence of stroke on cervical ausculation signals, but noticed certain discrepancies with regards to bolus viscosity.

**Conclusions:**

Overall, our results support the necessity of using multiple statistical features concurrently to identify laryngeal penetration of swallowed boluses in future work with high resolution cervical auscultation.

## Background

Oropharyngeal swallowing is produced through a complex series of short duration sensorimotor events that begin in the oral cavity and end in the esophagus. Swallowing activity modulates the alternation of the shared upper aerodigestive tract’s respiratory and digestive functions. The pharynx, which divides into the airway anteriorly and the esophagus posteriorly, is the conduit of both air to the lungs and food to the esophagus. The act of swallowing produces important valving actions that momentarily close the airway at the level of the larynx and open the upper esophagus, thereby delivering food to the digestive system and diverting it away from the lungs [1]. Impaired swallowing function, then, leads to either the ineffective transfer of food and liquids into the digestive system or to the misdirection of this same food and liquids into the respiratory system.

Oropharyngeal dysphagia (OPD) is one such impairment of the upper aerodigestive tract that disrupts the normal transfer of food and liquids from the mouth to the digestive system. Aspiration, the passage of gravity-dependent solid or liquid matter into the trachea, is recognized as the most clinically salient feature of OPD [2–5]. This can lead to immediate adverse outcomes such as airway obstruction as well as more insidious and long-term sequelae like aspiration pneumonia (AP) [2–5]. The incidence of AP in patients with documented OPD ranges from 11 to 22 %, and epidemiological investigations using hospital discharge summary data from the US Medicare system have identified a rising incidence of AP [6].

In its classical, familiar form as in a public place in which meals are served, aspiration by healthy people [7] is accompanied by choking and coughing. In the extreme scenario, this can lead to airway obstructions that remain a major cause of accidental death if not cleared by a Heimlich maneuver or other emergency procedure. In frail, immunologically or medically compromised people, and those with diseases that directly cause dysphagia by damaging the sensorimotor substrates enabling swallowing, aspiration can be completely undetectable (silent). This is the result of airway protective reflexes being attenuated or disconnected due to their underlying condition. Silent aspiration of saliva, typically mixed with the normal and pathological bacteria residing in the oral cavity [8], is a known cause of aspiration pneumonia which constitutes up to 15.5 % of all pneumonias [6]. As in all pneumonia scenarios, reversal of the cause of pulmonary inoculation by pathogens is the primary goal of medical treatment of aspiration pneumonia but these efforts cannot begin unless aspiration is detected. Screening and clinical assessment for oropharyngeal dysphagia and prandial (swallowing-related) aspiration is routinely conducted when there is a reasonable suspicion that dysphagia may be a potential source of a preventable pulmonary adverse event. This typically occurs after a patient develops a new onset of a condition known to cause OPD. These procedures rely on a review of the case history, assessment of oral-facial-pharyngeal sensorimotor function, and the observation of the patient swallowing (typically water). Definitive signs of likely prandial aspiration include coughing after or during swallowing, however some disease states disrupt the protective airway reflexes such as coughing and lead to asymptomatic or silent aspiration. The absence of clinical signs of aspiration alone do not justify a suspicion of ‘risk of silent aspiration’ because healthy people regularly swallow without signs of aspiration. However if coughing or signs of aspiration are absent and there are additional case-related factors that justify a suspicion of silent aspiration, then imaging tests such as the Videofluoroscopic Swallowing Study (VFSS) or Fiberoptic Endoscopic Evaluation of Swallowing (FEES) are routinely used to evaluate the kinematic functions of the aerodigestive tract and to assess airway protection during swallowing.

Due to the risk posed by aspiration, and subsequently pneumonia, early identification of aspiration, particularly silent aspiration, would be of great human importance and benefit. Dysphagia screening is a ubiquitous process that is performed on at-risk patients at their point of entry to a hospital or emergency room. It depends on the detection of aspiration signs by human observers and can include distinct actions such as spontaneous coughs, altered gag reflexes, or impaired tongue and oral motor performance as well as more subjective qualities such as vocalization modifications [9, 10]. Formal dysphagia screening protocols all include several case history and clinical examination factors in their pass-fail algorithms as well [11, 12], however formal protocols are not deployed in all institutions or settings [13]. In these situations where fewer factors are included in the patient’s assessment, the absence of cough in otherwise at-risk persons is more likely to lead to a false-negative identification of aspiration risk. Therefore those aspirators without overt signs of aspiration ‘pass’ screening tests and develop life-threatening adverse events [3, 6]. Early detection of silent aspiration at the moment the patient enters the health care system is crucial to lowering the morbidity associated with dysphagia, which has been estimated to be 50 % or greater for patients with a stroke [14–17]. Fortunately, instrumentation-based methods of assessment such as fiberoptic endoscopy and pulse oximetry have shown promise in raising the predictive accuracy of dysphagia screening [16, 17].

Cervical auscultation, a procedure traditionally performed with stethoscopes, has been an attractive addition to dysphagia screening but studies have found its predictive value to be poor, and its validity in identifying the presence of dysphagia, unsupported [18, 19]. High resolution cervical auscultation (HRCA) on the other hand, which uses digital microphones and accelerometers to detect vibrations and sounds caused by movement of the aerodigestive structures and swallowed material, has recently shown some promise in the detection of aspiration in patients with dysphagia [20–22]. Recent investigations with these HRCA devices have shown increasing accuracy in detecting specific swallowing events and in grossly differentiating between swallows that contain unsafe airway invasion of swallowed material [23].

The small size of HRCA instrumentation and non-invasive nature its deployment would allow for constant monitoring of a patient and could theoretically be used to detect when aspiration occurs in any situation. While some effort has been put towards detecting aspiration in the context of cervical auscultation, research in patients with dysphagia using HRCA to detect aspiration has been limited. The majority of such studies focused solely on normal swallows from healthy subjects and those that did not often had limited scope or small sample sizes [24].

This study seeks to provide a more generalized and widely applicable summary of cervical auscultation and its ability to differentiate safe and unsafe swallows. Our work has been organized similarly to our previous study, where we compared safe swallows made by both healthy and unhealthy subjects (Dudik, JM, Kurosu, A, Coyle, JL, Sejdić, E: The effects of dysphagia on swallowing sounds and vibrations in adults, under review). First, we simultaneously recorded sounds and vibrations of swallows that resulted in or carried the risk of deep laryngeal penetration or aspiration made by subjects with dysphagia (unsafe airway protection). We characterized these swallows through a number of time, frequency, and time-frequency features. Second, we statistically compared these values to those corresponding to swallows that did not carry a risk of deep laryngeal penetration or aspiration made by the same subjects (safe airway protection). Based on past studies which considered the same task, we believed that swallows with safe airway protection would differ from those with unsafe airway protection and that such differences would be detectable via cervical auscultation [19, 25, 26]. Such a direct comparison of statistical features would be able to clearly demonstrate the validity of that hypothesis. Past studies have also demonstrated how the patient’s sex and the viscosity of the bolus affect cervical auscultation signals, so the independent effects of these variables were also analyzed in this study to determine if their effects remained constant [27, 28]. Finally, we considered how cervical auscultation signals are affected by dysphagia as a result of stroke compared to other causes to determine if the most common cause of dysphagia presents any consistent cervical auscultation patterns that indicate common, underlying symptoms [14].

## Methods

Our data collection protocol, signal processing steps, and feature extraction techniques are all identical to our previous work with non-aspirating dysphagic subjects (Dudik, JM, Kurosu, A, Coyle, JL, Sejdić, E: The effects of dysphagia on swallowing sounds and vibrations in adults, under review). For completeness, the entire process is included below with minor changes to the description of our experimental groups. The protocol for the study was approve by the Institutional Review Board at the University of Pittsburgh.

### Data collection

Our recording equipment consisted of a tri-axial accelerometer and a contact microphone attached to the participant’s anterior neck with double-sided tape. The accelerometer (ADXL 327, Analog Devices, Norwood, Massachusetts) was mounted in a custom plastic case, and affixed over the cricoid cartilage as previously described in order to provide the highest signal quality [29]. The main accelerometer axes were aligned approximately parallel to the cervical spine and perpendicular to the coronal plane and will be referred to as the superior-inferior and anterior-posterior axes, respectively. The third axis was not used for this study as a comparable signal was not used in our study of healthy subjects [27]. The sensor was powered by a power supply (model 1504, BK Precision, Yorba Linda, California) with a 3V output, and the resulting signals were bandpass filtered from 0.1 to 3000 Hz with ten times amplification (model P55, Grass Technologies, Warwick, Rhode Island). The voltage signals for each axis of the accelerometer were both fed into a National Instruments 6210 DAQ and recorded at 20 kHz by the LabView program Signal Express (National Instruments, Austin, Texas). This setup has been shown to be effective at detecting swallowing activity in previous studies [23, 30]. The microphone (model C 411L, AKG, Vienna, Austria) was placed below the accelerometer and slightly towards the right lateral side of the trachea so as to avoid contact between the two sensors and prevent obstruction of the radiographic view of the upper airway, but still record events from approximately the same location. This location has previously been described to be appropriate for collecting swallowing sound signals [29, 31]. The microphone was powered by a power supply (model B29L, AKG, Vienna, Austria) and set to ‘line’ impedance with a volume of ‘9’ while the resulting voltage signal was sent to the previously mentioned DAQ. This signal was left unfiltered, as an upper limit to the bandwidth of swallowing sounds has not yet been found. The signal was sampled by Signal Express at 20 kHz. These sensors were attached before and allowed to collect data during a videofluoroscopic swallowing assessment, so concurrent videofluoroscopy images were also obtained. The images output by the x-ray machine (Ultimax system, Toshiba, Tustin, CA) were input to a video capture card (AccuStream Express HD, Foresight Imaging, Chelmsford, MA) and recorded with the same Labview program.

A total of 76 patients with suspected dysphagia that were scheduled to undergo a videofluoroscopic swallowing evaluation at the University of Pittsburgh Medical Center (Pittsburgh, Pennsylvania) served as the sample. Participants were recruited from the general inpatient and outpatient population of persons referred to the Speech Language Pathology service for instrumental assessment of oropharyngeal swallowing function with videofluoroscopy (VFS). As a result of the high prevalence of multiple comorbidities in patients with dysphagia and the interactions of these conditions is causing dysphagia, there were few patients for whom a single admitting or hospital-acquired diagnosis could be pinpointed as the sole cause of their dysphagia. Among the most common diagnoses in our cohort were stroke (17), organ transplantation (13 lung, 3 heart, liver, renal or multiple organs), dysphagia not otherwise specified (19), respiratory failure (7), non-stroke neurological disease (6), cancer - lung, esophageal, head-neck (3), and pneumonia (8). A total of 17 patients (10 men, 7 women, mean age 67) had a current diagnosis of stroke while the remaining 59 (40 men, 19 women, mean age 61) had medical conditions unrelated to stroke. Those patients that had a history of major head or neck surgery, were equipped assistive devices that obstructed the anterior neck such as a tracheostomy tube, or were not sufficiently competent to give informed consent were not included in the study, but no other conditions were excluded. Patients with dysphagia did not undergo a standardized data collection procedure, as the videofluoroscopy examination is routinely modified by the examiner to suit the individual patient. This method of data acquisition more closely represents the actual clinical environment. All analyzed swallows were limited to those made while the participant’ head was in a neutral head position. Swallows made with maneuvers such as the effortful swallow, supraglottic swallow, or Mendelsohn maneuver were also excluded. The liquids swallowed during the examination included chilled (5 °C) Varibar Thin Liquid, with <5 cps consistency, and Varibar Nectar, with ≈300 cps consistency, (Bracco, Milan, ITA) presented as either self-administered from a cup in comfortable volumes self-selected by the patient, or administered by the examiner in volumes of approximately 3 mL from a 5 mL spoon. A total of 468 swallows (128 from patients with stroke, 340 without) had no more than minor penetration of the bolus into the larynx while 53 swallows (19 from those with stroke, 34 without) had greater penetration or residue. These groups can be classified as having a Penetration Aspiration-score of 3 or less in the first group or a score of 4 or greater in the second, the importance of which is explained in the following section [32, 33].

### Signal processing and analysis

Data recorded with the accelerometer underwent several processing steps to improve its signal quality. A signal recorded from the device when presented with no input on a previous date was used to generate an auto-regressive model of the device’s noise. The coefficients of this model were then used to generate a finite impulse response filter that was used to remove the device noise from the recorded signal. Afterwards, motion artifacts and other low frequency noise were removed from the signal through the use of least-square splines. Specifically, we used fourth-order splines with a number of knots equal to $\frac {\text {\textit {Nf}}_{l}}{f_{s}}$, where *N* is the number of data points in the sample, *fs* is the original 10 kHz sampling frequency of our data, and *f*_*l*_ is equal to either 3.77 or 1.67 Hz for the superior-inferior or anterior-posterior direction, respectively. The values for *f*_*l*_ were calculated and optimized in previous studies. Finally, we attempted to minimize the impact of broadband noise on the signal by utilizing wavelet denoising techniques. Specifically, we chose to use tenth-order Meyer wavelets with soft thresholding. The value of our threshold was chosen to equal $\sigma \sqrt {2\log N}$, where *N* is the number of samples in the data set and *σ*, the estimated standard deviation of the noise, is defined as the median of the down-sampled wavelet coefficients divided by 0.6745. We applied the same FIR filtering and wavelet denoising techniques to the microphone signal after re-calculating the appropriate coefficients. No splines or other low-frequency removal techniques were applied to the swallowing sounds because we had not investigated if such frequencies contained important sound information.

Two judges, both speech language pathologists with dysphagia research experience and whose inter- and intra-rater reliability in the measures used in this study have been established in prior published research, visually inspected the fluoroscopic data to measure two parameters: the duration of the swallowing segments and the extent of airway penetration or aspiration during the swallowing segments using the penetration aspiration scale [32]. One of these judges is a co-developer of the penetration aspiration scale who developed decision-making rules for selection of specific frames marking segment duration onset and offset and in rating of the extent of airway protection during the swallow using the eight-point penetration-aspiration scale. They then trained the second judge in methods of selection of these video frames. After training, both judges evaluated a set of twenty-five unfamiliar video recorded swallows, none of which were included in the participant data for the present study. Judgment reliability was evaluated using the intraclass correlation coefficient. The intra-rater and inter-rater intraclass correlation coefficients were both 0.998. Following establishment of acceptable intra- and inter-rater reliability for segment durations and penetration-aspiration scores, the second judge then evaluated the segment onset, segment offset, and penetration-aspiration scale scores for each swallow described in the present study.

Blinded to the accelerometry data, these judges segmented and labelled each individual swallow. The beginning (onset) of a swallow segment was defined as the time at which the leading edge of the swallowed bolus intersected with the shadow cast on the x-ray image by the posterior border of the ramus of the mandible while the end (offset) was the time at which the hyoid bone completed motion associated with swallowing-related pharyngeal activity and returned to its resting or pre-swallow position. The time points provided by this procedure were used to segment the vibratory and acoustic signals, thereby obtaining individual swallow data. Each swallow was also rated on a standard 8-point ordinal clinical penetration-aspiration scale (PA scale) [32] and any swallows with a rating of 3 or lower was included in our analysis as a non-aspirating swallow. Scores of 3 or lower on this scale indicate that either no material entered the upper airway (score of 1), or shallow penetration of the larynx without (score of 2) or with (score of 3) some residue of swallowed material remaining in the larynx after the swallow. This cutoff point for safe-unsafe scores as chosen because deeper laryngeal penetration, and especially aspiration into the trachea, represented by scale scores of 4 and higher, have been found to occur with negligible frequency in healthy persons, and for the purposes of our study, were considered to be ‘unsafe’ swallows. These PA scores were then compared to signals acquired through the cervical auscultation devices [33, 34].

Once the auscultation signals were filtered and segmented we calculated several different features in order to characterize each swallow. In the time domain, we investigated the skewness and kurtosis of the signal, which can be calculated with the typical statistical formulas [35]. We also calculated multiple information-theoretic features by following the procedure outlined in previous publications. The signals were normalized to zero mean and unit variance, then divided into ten equally spaced levels, ranging from zero to nine, that contained all recorded signal values. We then calculated the entropy rate feature of the signals. This is found by subtracting the minimum value of the normalized entropy rate of the signal from 1 to produce a value that ranges from zero, for a completely random signal, to one, for a completely regular signal [23]. The normalized entropy rate is calculated as

(1) $$ NER(L)=\frac{SE(L)-SE(L-1)+SE(1)*perc(L)}{SE(1)}  $$

where *perc* is the percent of unique entries in the given sequence *L* [23]. *SE* is the Shannon entropy of the sequence and is calculated as

(2) $$ SE(L)=-\sum\limits_{j=0}^{10^{L}-1}\rho(j)\ln(\rho(j))  $$

where *ρ*(*j*) is the probability mass function of the given sequence. Quantizing the original signal to 100 discrete levels instead of ten allowed us to calculate the Lempel-Ziv complexity as

(3) $$ C=\frac{k\log_{100}n}{n}  $$

where *k* is the number of unique sequences in the decomposed signal and *n* is the pattern length [36].

We also investigated several features in the frequency domain. The center frequency, sometimes referred to as the spectral centroid, was simply calculated by taking the Fourier transform of the signal and finding the weighted average of all the positive frequency components:

(4) $$ C = \frac{\sum\limits_{n=0}^{N-1} f(n)x(n)}{\sum\limits_{n=0}^{N-1}x(n)}  $$

where *x*(*n*) is the magnitude of a frequency component and *f*(*n*) is the frequency of that component. Similarly, the peak frequency was found to be the Fourier frequency component with the greatest spectral energy. We defined the bandwidth of the signal as the standard deviation of its Fourier transform [23].

Lastly, we characterized our signal in the time-frequency domain. Previous contributions found that swallowing signals are to some degree non-stationary [37], to which wavelet decomposition is better suited than a simple Fourier analysis [38–40]. We chose to decompose our signal using tenth-order Meyer wavelets because they are continuous, have a known scaling function [41, 42], and more closely resemble swallowing signals in the time domain compared to Gaussian or other common wavelet shapes [43]. The energy in a given decomposition level was defined as

(5) $$ E_{x}=||x||^{2}  $$

where *x* represents a vector of the approximation coefficients or one of the vectors representing the detail coefficients. ||∗|| denotes the Euclidean norm [23]. The total energy of the signal is simply the sum of the energy at each decomposition level. From there, we could calculate the wavelet entropy as:

(6) $$ WE = -\frac{Er_{a_{10}}}{100} \log_{2}{\frac{Er_{a_{10}}}{100}} -\sum\limits_{k=1}^{10} \frac{Er_{d_{k}}}{100} \log_{2}{\frac{Er_{d_{k}}}{100}}  $$

where *Er* is the relative contribution of a given decomposition level to the total energy in the signal and is given as [23]

(7) $$ Er_{x}=\frac{E_{x}}{E_{total}}*100\,\%  $$

### Statistical analysis

After calculating the relevant features we performed various statistical comparisons on our data set. First, we attempted to test for the normality of our data with the Shapiro-Wilk test as well as the equality of variances via the Levene’s test in order to assess the viability of using parametric tests. However, after separating the data based on our chosen variables (PA score, participant’s sex, presence of stroke, bolus viscosity) we found that approximately 60 % of our feature distributions met these assumptions. At this point, we chose to incorporate non-parametric tests to analyze our data.

We used the Wilcoxon signed rank test to identify differences with regards to each feature of all three signals for safe (PA scores of 1–3) and unsafe (PA scores of 4–8) swallows and stratified by the consistency of the ingested bolus. A p-value of ≤0.05 was used to determine significance. This process was repeated to test for differences between dysphagic patients with and without stroke during ‘unsafe’ swallows. To mirror the results of our previous studies we performed another set of rank sum tests to examine sex-based differences in the signals recorded from the dysphagic population. Finally, the effects of bolus viscosity on our data was examined through the use of Wilcoxon signed-rank tests. The age of the subjects was not utilized as a variable since previous work has shown little significant effect of age on cervical auscultation signals even for large age differences [28].

Post hoc estimates of our statistical power were carried out in the GPower software program [44]. We used Lehmann’s method of estimation with a target power of at least 0.80. In mathematical form:

(8) $$ power = 1-\Phi \left(\frac{c-E(W)}{\sqrt{Var(W)}}\right)  $$

where *c* is the critical value of the test statistic and is equal to 1.64, *E*() and *Var*() are the expected value and variance operators, respectively, and *Φ* is the normal cumulative distribution function. *W* is the Mann-Whitney statistic and is the number of instances where a data point from one group has a lower rank than the data points in the alternate group. With small variations between them due to the variable population sizes, we found that our comparisons had sufficient power to differentiate moderately sized (*d*=0.40±0.05) effects.

## Results

**Fig. 1:**
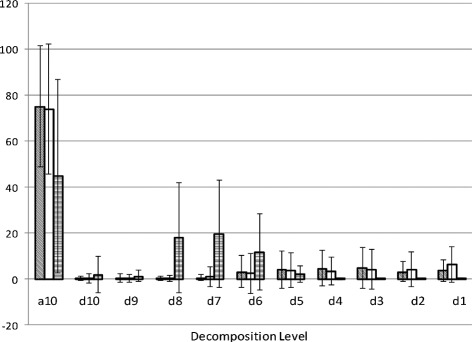
Wavelet Energy Distributions. Wavelet energy composition of swallowing vibrations and sounds during an unsafe swallow. From left to right, the bars for each decomposition level correspond to the signals recorded from the anterior-posterior accelerometer (*diagonal lines*), the superior-inferior accelerometer (*white*), and the microphone (*horizontal lines*)

Tables 1, 2, 3 present the mean and interquartile range of each feature of our data set separated by bolus viscosity and whether it was a safe or unsafe swallow. Figure 1 displays the average wavelet decomposition of all three of our signals corresponding to unsafe swallows.

**Table 1:** Feature values corresponding to anterior-posterior swallowing vibrations

	Thin		Viscous	
	Safe	Unsafe	Safe	Unsafe
Skewness	0.867, 1.470	0.642, 1.963	0.491, 1.455	0.759, 1.621
Kurtosis	87.04, 25.48	27.56, 29.84	96.20, 30.07	39.69, 15.85
Entropy Rate	0.987, 0.007	0.987, 0.007	0.989, 0.006	0.986, 0.004
L-Z Complexity	0.059, 0.031	0.065, 0.030	0.056, 0.039	0.064, 0.029
Peak Freq (Hz)	16.56, 4.732	7.162, 5.161	56.29, 4.414	15.22, 5.613
Center Freq (Hz)	189.7, 138.3	109.2, 115.4	204.7, 113.4	141.0, 78.93
Bandwidth (Hz)	221.1, 222.6	198.1, 158.2	273.7, 174.7	264.2, 86.94
Wavelet Entropy	1.034, 1.348	1.003, 0.976	0.928, 1.139	1.066, 1.120

**Table 2:** Feature values corresponding to superior-inferior swallowing vibrations

	Thin		Viscous	
	Safe	Unsafe	Safe	Unsafe
Skewness	–0.557, 1.082	–0.435, 2.305	–0.129, 1.207	–0.441, 0.754
Kurtosis	28.91, 14.79	101.6, 31.11	66.26, 11.13	22.68, 8.000
Entropy Rate	0.988, 0.005	0.989, 0.003	0.989, 0.006	0.988, 0.004
L-Z Complexity	0.068, 0.033	0.067, 0.032	0.062, 0.038	0.069, 0.032
Peak Freq (Hz)	11.33, 3.660	19.52, 7.336	10.79, 3.627	30.60, 3.812
Center Freq (Hz)	67.53, 47.45	143.4, 56.31	105.7, 44.94	85.52, 20.85
Bandwidth (Hz)	114.7, 99.64	238.4, 62.16	145.3, 107.5	180.4, 35.51
Wavelet Entropy	1.160, 1.178	0.978, 0.996	1.138, 1.001	1.004, 1.408

**Table 3:** Feature values corresponding to swallowing sounds

	Thin		Viscous	
	Safe	Unsafe	Safe	Unsafe
Skewness	–0.317, 3.334	–1.564, 3.531	–0.525, 3.894	–0.125, 3.873
Kurtosis	149.2, 117.0	187.2, 101.0	191.8, 132.4	157.3, 88.60
Entropy Rate	0.985, 0.009	0.986, 0.009	0.987, 0.007	0.987, 0.007
L-Z Complexity	0.055, 0.031	0.055, 0.048	0.050, 0.026	0.052, 0.038
Peak Freq (Hz)	94.10, 122.3	99.52, 77.71	99.46, 130.4	92.88, 100.2
Center Freq (Hz)	312.5, 315.9	348.5, 245.7	340.3, 305.1	382.2, 294.2
Bandwidth (Hz)	348.2, 281.8	393.6, 170.0	402.7, 284.0	399.0, 230.7
Wavelet Entropy	1.723, 1.151	1.641, 1.119	1.596, 0.946	1.697, 1.615

We found no significant differences in any of our features for safe or unsafe thin liquid swallows. For viscous swallows, we found that the anterior-posterior vibrations had greater Lempel-Ziv complexities (*p*=0.039) and lower entropy rates (*p*=0.022) during unsafe swallows. We also found that the superior-inferior accelerometer bandwidth was greater for unsafe swallows (*p*=0.033), while the microphone peak frequency was lower (*p*=0.048) when compared to safe swallows.

Our contrasts with regards to bolus viscosity and the presence or absence of stroke showed no significant effects of either variable on unsafe swallows. However, we did note several differences with regards to patient sex. Specifically, we found that unsafe swallows made by male subjects showed greater anterior-posterior kurtosis (*p*=0.013) and superior-inferior Lempel-Ziv complexity (*p*=0.016) corresponding to vibrations along with greater entropy rate (*p*=0.015), center frequency (*p*=0.045), and bandwidth (*p*=0.047) corresponding to swallowing sounds.

## Discussion

We found that HRCA is able to detect several statistical differences between unsafe swallows of viscous fluid, in which clinically significant aspiration and laryngeal penetration occurred, and safe swallows that either exhibited no airway penetration or airway penetration that falls within the normal range for healthy people. This is of particular interest because aspiration of thicker liquids has been shown to produce higher rates of pneumonia than aspiration of thin liquids, and longer hospitalization durations than those observed in aspirators drinking thinner liquids [5]. As cervical auscultation signals are not fully understood, we postulate the reasons for why only viscous swallows demonstrated significant differences in this situation. Past research has suggested that thickening agents used during videofluoroscopy exams exhibit non-Newtonian fluid properties, which lead to the reduced aspiration rate in dysphagic patients [45, 46]. It is possible that the penetration of this non-Newtonian fluid into the airway affects the recorded signals in ways that do not occur during thin or non-aspirating viscous swallows. For example, a sudden drop in the pressure exerted on the aspirated material as it enters the larynx could notably reduce the viscosity, and subsequently change the acoustic properties, of a viscous bolus while a thin bolus would be unaffected. Alternatively, viscous swallows are used in the clinical setting because, among other reasons, they provide greater feedback to the patient during a swallow [47, 48]. Whether consciously or unconsciously, it is possible that the patient is better able to determine when swallowed material has entered the larynx and react accordingly when aspirating viscous material. This physiological change could alter the cervical auscultation signals as demonstrated in this study.

It is also interesting to note that, when compared to their values for safe swallows, the values of many of the features corresponding to unsafe swallows are closer to the values found in a previous study corresponding to safe swallows made by healthy subjects [28]. It may be that our data indicates that deep laryngeal penetration or aspiration occurs when a subject with reduced airway protection performs a swallow as if they did not have a swallowing impairment. In this situation, the patient with dysphagia would behave identically to a healthy subject except for one small detail, such as delaying epiglottic inversion, that would allow material to enter the larynx. A patient that had dysphagia but swallowed safely may have developed a modified swallowing profile that compensates for their specific deficiency of airway protection. A similar but alternative explanation is that cervical auscultation is unable to detect the occurrence of aspiration itself, but instead is able to monitor the activity of related swallowing events. As an example, we can imagine a situation where we have patient with dysphagia due to delayed epiglottic inversion and our sensors can record the sounds and vibrations made by the bolus as it travels through the pharynx, but not the larynx. If the patient does execute an unsafe swallow, then it may be because the bolus was travelling as it normally would in a person with full airway protection. On the other hand, if the patient executes a safe swallow it may be because of a longer than normal bolus transit time, which would allow for full airway protection in spite of the inversion delay. In this situation our sensors would be able to identify the abnormal swallowing pattern of the safe swallow, but the unsafe swallow would demonstrate little difference from a normal, healthy subject. This distinction between aspiration and altered swallowing patterns could be a vital detail in future work, since aspiration is more common among, but not exclusive to, patients with dysphagia. However, many more statistical features and physiological events would need to be investigated in order to reach a proper consensus on any of these topics, which is beyond the scope of the current manuscript.

Lastly, our sex-based contrasts match our previous work (Dudik, JM, Kurosu, A, Coyle, JL, Sejdić, E: The effects of dysphagia on swallowing sounds and vibrations in adults, under review) and [28], with males demonstrating higher frequency components and greater kurtosis than female counterparts. As described in those studies, we suggest that this is a result of the physical differences of the laryngeal prominence and that future studies should account for these differences during classification tasks. Fewer features showed statistical significance in this regard, however, which we believe to be a result of the added effects of dysphagia and poor airway protection as confounding variables.

Much past work has focused on classifying whether airway protection during swallowing was safe or unsafe, rather than directly characterizing unsafe swallows [21, 49–52]. However in order to achieve the reported accuracies, these classification techniques simultaneously utilize multiple features that were selected either through principle component analysis [49, 51] or because the features were of particular interest to the researcher [21, 50, 52]. All of these studies found that using at least two features [21], if not more [49–52], provided noticeable improvement of the data classification when compared to using the value of a single signal feature. Our findings demonstrate the reason for these findings. Though our feature value distributions are not identical between safe and unsafe swallows, we were able to find very few significant differences between individual features for the two states. Attempting to classify swallows using only a single, generalized statistical feature would produce mediocre results at best. This is not to say that all of our chosen features would be useful for such a task, but that future research into classifying unsafe swallows would need to investigate the concurrent predictive value of their statistical features.

These results come with three key limitations, however. First, it is possible that the effects of deep laryngeal penetration and aspiration on swallowing sounds and vibrations were masked or attenuated by other variables. Dysphagia is a highly varied condition that may take completely different forms between patients with the same diagnosis or even between individual swallows from the same patient. Our previous study as well as the work of others showed that safe swallows made by healthy subjects and dysphagic patients showed multiple statistical differences between, but relatively high variation of, individual feature values [19, 25, 26] and (Dudik, JM, Kurosu, A, Coyle, JL, Sejdić, E: The effects of dysphagia on swallowing sounds and vibrations in adults, under review). This study demonstrated that features corresponding to unsafe swallows are similarly variable. As mentioned previously, it is possible that the main source of cervical auscultation signals is not the deep laryngeal penetration and aspiration event itself, but other swallowing events that may be altered in these patients. Second, our lack of any notable statistical differences between unsafe swallows made by subjects with or without stroke matches our findings with respect to safe swallows (Dudik, JM, Kurosu, A, Coyle, JL, Sejdić, E: The effects of dysphagia on swallowing sounds and vibrations in adults, under review). It is possible that our findings indicate that there is not a single consistent physiological expression of dysphagia as a result of stroke, but may also demonstrate that cervical auscultation is unable to identify key existing features of dysphagia caused by a stroke. In either case, this demonstrates that additional investigations will need to be done to characterize the most common form of dysphagia before classification methods could be fully implemented. Finally, our results indicate that cervical auscultation can more easily identify unsafe viscous swallows than unsafe thin swallows. Since aspirating with thin boluses is more common and occurs more often outside of the clinical environment this may restrict the number of potential applications for cervical auscultation. However, we only utilized a small selection of very generalized statistical features in this study. A follow-up study that utilizes features more focused towards cervical auscultation signals or a full machine-learning study could provide a better estimate of the technique’s usefulness.

## Conclusion

In this study, we recorded swallowing sounds and vibrations from adult patients with dysphagia who exhibited either deep laryngeal penetration or aspirated on one or more swallows during a routine videofluoroscopy exam. We found only a very limited number of statistical differences between swallows during which deep laryngeal penetration or aspiration (unsafe swallows) and those during which only shallow or no laryngeal penetration occurred (safe swallows) based on our chosen features. This supports the findings of other studies and demonstrates the necessity of utilizing multiple statistical features to characterize aspiration. We suggest that the difference we did find is due to a complex interaction between the non-Newtonian nature of thickened liquids and the reduced airway protection in dysphagic patients. We also confirmed the findings of our earlier work with regards to the effects of stroke and sex on cervical auscultation signals. In summary, we conclude that no simple statistical feature can be used to characterize impaired airway protection in dysphagic patients, and that multiple features must be accounted for when aspiration is chosen as a variable in future work.
